# Amygdalar Functional Connectivity Differences Associated With Reduced Pain Intensity in Pediatric Peripheral Neuropathic Pain

**DOI:** 10.3389/fpain.2022.918766

**Published:** 2022-05-27

**Authors:** Madeleine Verriotis, Clarissa Sorger, Judy Peters, Lizbeth J. Ayoub, Kiran K. Seunarine, Chris A. Clark, Suellen M. Walker, Massieh Moayedi

**Affiliations:** ^1^Paediatric Pain Research Group, Developmental Neurosciences Department, UCL Great Ormond Street Institute of Child Health, London, United Kingdom; ^2^Department of Anaesthesia and Pain Medicine, Great Ormond Street Hospital NHS Foundation Trust, London, United Kingdom; ^3^Centre for Multimodal Sensorimotor and Pain Research, University of Toronto, Toronto, ON, Canada; ^4^Faculty of Dentistry, University of Toronto, Toronto, ON, Canada; ^5^University of Toronto Centre for the Study of Pain, Toronto, ON, Canada; ^6^Division of Clinical and Computational Neuroscience, Krembil Brain Institute, Toronto Western Hospital, University Health Network, Toronto, ON, Canada; ^7^Developmental Imaging and Biophysics Section, Developmental Neurosciences Department, UCL Great Ormond Street Institute of Child Health, London, United Kingdom

**Keywords:** chronic pain, neuropathic pain, functional connectivity, fMRI, limbic, brain, children, teenager

## Abstract

**Background:**

There is evidence of altered corticolimbic circuitry in adults with chronic pain, but relatively little is known of functional brain mechanisms in adolescents with neuropathic pain (NeuP). Pediatric NeuP is etiologically and phenotypically different from NeuP in adults, highlighting the need for pediatric-focused research. The amygdala is a key limbic region with important roles in the emotional-affective dimension of pain and in pain modulation.

**Objective:**

To investigate amygdalar resting state functional connectivity (rsFC) in adolescents with NeuP.

**Methods:**

This cross-sectional observational cohort study compared resting state functional MRI scans in adolescents aged 11–18 years with clinical features of chronic peripheral NeuP (*n* = 17), recruited from a tertiary clinic, relative to healthy adolescents (*n* = 17). We performed seed-to-voxel whole-brain rsFC analysis of the bilateral amygdalae. Next, we performed *post hoc* exploratory correlations with clinical variables to further explain rsFC differences.

**Results:**

Adolescents with NeuP had stronger negative rsFC between right amygdala and right dorsolateral prefrontal cortex (dlPFC) and stronger positive rsFC between right amygdala and left angular gyrus (AG), compared to controls (P_*FDR*_<0.025). Furthermore, lower pain intensity correlated with stronger negative amygdala-dlPFC rsFC in males (*r* = 0.67, *P* = 0.034, *n* = 10), and with stronger positive amygdala-AG rsFC in females (*r* = −0.90, *P* = 0.006, *n* = 7). These amygdalar rsFC differences may thus be pain inhibitory.

**Conclusions:**

Consistent with the considerable affective and cognitive factors reported in a larger cohort, there are rsFC differences in limbic pain modulatory circuits in adolescents with NeuP. Findings also highlight the need for assessing sex-dependent brain mechanisms in future studies, where possible.

## Introduction

Chronic neuropathic pain (NeuP) in children and adolescents can be severe and difficult to manage (1, 2). Differing causes of NeuP in adolescents compared to adults (1), and laboratory studies indicating that pain mechanisms change with age (3), highlight the need for pediatric-focused research. Structural and functional neuroimaging studies in adults have improved understanding of mechanisms associated with chronic pain (4, 5), and highlighted the importance of altered corticolimbic circuitry in chronic pain (6, 7). Neuroimaging studies have evaluated chronic pain in pediatric cohorts (8–10), with many focussed on complex regional pain syndrome (CRPS) (11–17), but relatively little is known of central neural mechanisms in pediatric peripheral NeuP. Common causes of NeuP in children include lesion (e.g., trauma, surgery, chemotherapy) or disease (e.g., genetic, neurological) of the somatosensory nervous system (18). Typical features include specific descriptors of pain (e.g., burning/hot, electric shocks/shooting, pricking/pins and needles) and symptoms of allodynia, dysaesthesia, or sensory loss, and a biopsychosocial approach is required for management (18, 19).

The amygdala is a key limbic region with important roles in the emotional-affective dimension of pain and in pain modulation (20–22). Experimental noxious stimuli have elicited greater amygdalar activity in both healthy adults and those with clinical pain conditions, while fewer studies identified decreases in amygdala activity (23). A shift in pain processing from sensory to emotion-related circuitry, including the amygdala, has also been reported in the transition from subacute to chronic back pain (24). There is now increased emphasis on brain network connectivity in pain neuroimaging studies (4), including resting-state functional connectivity (rsFC), to investigate the intrinsic architecture of the brain and abnormalities in chronic pain (8, 25–27). Studies of adults with chronic pain report increased amygdala rsFC with corticolimbic regions associated with cognitive/affective control (anterior cingulate cortex), memory (parahippocampal gyrus), and sensory-discriminative (insula, thalamus, sensorimotor cortices) processing, and with the frontoparietal network, compared to pain-free individuals (28–30). Similarly, abnormally increased amygdalar connectivity to multiple regions, including cognitive/affective (midcingulate cortex, prefrontal cortex), sensory-discriminative (thalamus, sensorimotor cortices), and memory-related regions, has been reported in adolescents with CRPS compared to pain-free adolescents, which normalized following treatment (13). Consistent with the sex-dependent prevalence of pediatric CRPS (31), this cohort was predominantly female (13). The amygdala is sensitive to sex hormone changes, especially in the developing brain during adolescence (32), but it is not known whether altered amygdalar connectivity in adolescents generalizes across both sexes, and across a range of pediatric chronic pain conditions including NeuP.

Pain experience is shaped by sensory and cognitive-affective factors. Pain catastrophizing is a measure of maladaptive cognitions that correlates with increased self-reported pain intensity and psychological distress (33–38). In adults with chronic pain, increased amygdala functional connectivity has been associated with both greater pain intensity (30) and pain catastrophizing (28). We found that adolescents with chronic peripheral NeuP pain reported moderate-severe intensity pain and high levels of pain catastrophizing (39), but associations with altered affective circuitry have not been assessed in this population.

Here, we focused on amygdala rsFC in both male and female adolescents with chronic peripheral NeuP. Given its role in pain modulation and cognitive-affective processing (20, 40), we hypothesized that amygdalar rsFC to nociceptive processing, cognitive-affective, or pain modulatory regions, such as the insular, cingulate, and prefrontal cortices (13, 28–30), would be different in adolescents with chronic NeuP compared to healthy controls. Our secondary hypothesis was that connectivity differences would correlate with pain intensity and/or pain catastrophizing. Exploratory *post hoc* analyses assessed potential sex differences, albeit in small subgroups. Increased understanding of potential mechanisms in understudied pediatric NeuP will allow more detailed phenotyping and tailored management.

## Methods

### Participants

Seventeen adolescents aged 11–18 years with a clinical diagnosis of chronic NeuP by a multidisciplinary team at the Great Ormond Street Hospital (GOSH) National Health Service (NHS) Foundation Trust Pain Clinic participated in this MRI study (Table 1). Adolescents were included if they reported NeuP symptoms in the limbs or torso and had quantitative sensory testing (QST) findings consistent with peripheral NeuP (39). Peripheral NeuP conditions included patients with peripheral neuropathy and distal neuropathic symptoms and signs or localized nerve lesion/injury (*n* = 7) and patients with persistent pos*t-*surgical pain and clinical and QST features of neuropathic pain in the region of prior surgery (*n* = 10). Adolescents were excluded if they had MRI contraindications or comorbid conditions, as described previously (41).

**Table 1:** Demographic characteristics of included participants with NeuP.

**Patient**	**Sex**	**Age (yrs)**	**Diagnosis**	**Pain site**	**Pain side**	**Pain duration**	**Medication**
1	M	15.4	PNP	lower leg	bilateral	>5 years	AD
2	M	12.9	PPSP	abdominal	bilateral	>5 years	SC, NB
3	M	16.9	PNP	lower leg	bilateral	>5 years	SC*
4	F	12.7	PNP	lower back	R	2–5 years	AC, SC
5	M	13.3	PPSP	lower leg	bilateral	2–5 years	AD, SC
6	F	16.7	PPSP	lower leg	R	2–5 years	AD, SC, NB
7	M	14.4	PPSP	upper leg	R	>5 years	AC, opioid+, SC
8	M	15.2	PNP	lower leg	R	2–5 years	AD, AC
9	F	16.7	PPSP	lower leg	R	2–5 years	none
10	F	17.4	PPSP	lower leg	L	2–5 years	opioid^∧^, SC
11	M	11.6	PNP	lower leg	R	1–2 years	none
12	F	16.6	PPSP	chest	L	>5 years	SC
13	M	15.5	PNP	lower leg	bilateral	>5 years	AD
14	F	16.9	PPSP	lower leg	bilateral	2–5 years	AC, AD
15	F	16.5	PNP	whole leg	L	1–2 years	AC
16	M	11.7	PPSP	lower leg	L	>5 years	SC
17	M	15.9	PPSP	lower leg	L	1–2 years	none

Medication includes only regular medication taken currently and excludes paracetamol and NSAIDs. The category “PNP” includes distal neuropathic symptoms and localized nerve lesion/injury. AC, anticonvulsant; AD, antidepressant; PNP, peripheral neuropathic pain; PPSP, persistent post-surgical pain; NB, nerve block; NeuP, neuropathic pain; opioid+, postoperative weaning morphine; opioid^∧^, PRN tramadol; SC, sodium channel blockers (lidocaine patch); SC*, oral mexiletine.

MRI participants were part of a larger cross-sectional observational cohort study that included evaluation of patien*t-* and paren*t-*reported outcome measures (PROMs) and QST (39) (https://clinicaltrials.gov/ct2/show/NCT03312881). Written informed parental consent and adolescent assent/consent were obtained (National Health Service Research Ethics Committee Approval 17/WM/0306; 23-8-2017). The MRI scan was performed within 3 months of participants' initial recruitment, and the acceptability and feasibility of research MRI in a different but overlapping sample of adolescents with chronic NeuP have been previously reported (41). Current data relate to recruitment and MRI scanning at GOSH from 19 October 2017 to 16 January 2020. Reporting is consistent with the STROBE (Strengthening the Reporting of Observational studies in Epidemiology) guidelines for cohort studies (42) and includes biological sex (male/female), as patients were not asked to self-report gender.

Patient data were compared with scans from 17 adolescents from an existing healthy control database at the UCL Great Ormond Street Institute of Child Health (UCL GOS ICH) (43), collected with the same MRI scanner, head coil, and scanning protocol. As part of the consent process for research MRI scans at GOSH and UCL GOS ICH, participants are given the option to consent to inclusion of their pseudonymised data in additional studies. The patient and control groups were age-matched, but there were fewer females in the patient group (Table 2).

**Table 2:** Comparative demographic data for patients and control participants.

	**NeuP (*n =* 17)**	**HC (*n =* 17)**	**Group comparisons**	**Effect size**
**Demographics**				
Age	15.5 (13.1–16.7) [11.6–17.4]	15.2 (13.2–16.3) [11.3–18.0]	*Z* = 0.48, *P =* 0.629	0.007
Male/Female (%F)	10/7 (41%)	6/11 (65%)	$\chi_{\left(1\right)}^{2}$ = 1.89, *P =* 0.303	0.236

*Data = median (25th-75th percentile) [min-max]. Effect size = η^2^ for Mann-Whitney test and Phi for χ^2^ test. Standardized test statistic (Z-value) is reported for Mann-Whitney test. F, female; HC, healthy control participants; NeuP, patients with neuropathic pain*.

### Pain Catastrophizing in Adolescents With NeuP

The Child version of the Pain Catastrophizing Scale (PCS-C) (44, 45) was completed as part of interdisciplinary assessment at pain clinic appointments in 15/17 patients and retrieved from the patient record (39). The PCS-C is a 13-item validated questionnaire that assesses 3 domains of pain cognitions: rumination, magnification, and helplessness (45). The maximum score is 52, with suggested clinical reference points of 0-14 for low, 15-25 for moderate, and ≥26 for high catastrophizing levels (46).

### Procedure

Participants attended a single study session for MRI scanning.

#### Pain Intensity Ratings in Adolescents With NeuP

Prior to the MRI scan, 12 participants reported average and worst pain intensity in the last week using a visual analog scale (VAS; 0–10 cm) (47). For 5 participants ratings were reported with the same scale at the time of recruitment (39).

#### MRI Data Acquisition

Participants were scanned on a 3T Siemens Prisma MRI scanner with a 64-channel head coil during allocated research scan sessions at GOSH. Neuroimaging included structural T1-weighted images, followed by resting-state functional MRI (rsfMRI). Scanning time was up to 30 min. Participants watched a movie of their choice during structural acquisitions; for the rsfMRI scan this was switched off and participants were asked to keep their eyes closed and let their minds wander.

A 3D T1-weighted scan was acquired using a magnetization prepared rapid gradient echo (MPRAGE) sequence with the following parameters: echo time (TE)/repetition time (TR) = 2.74/2,300ms; inversion time = 909 ms; 240 slices; flip angle = 8°; in-plane matrix resolution = 256 × 256 and field-of-view = 256 x 256 mm, resulting in a voxel size = 1 × 1 × 1mm; with a GRAPPA acceleration factor = 2.

A rsfMRI scan was acquired using a T2^*^-weighted echo-planar pulse imaging (EPI) sequence with the following parameters: TE/T*R* = 26/1,240 ms; 40 slices; flip angle = 75°; in-plane matrix resolutio*n* = 80 × 80 mm; field-of-view = 200 × 200 mm; voxel size = 2.5 × 2.5 × 2.5 mm; 300 volumes; with a multiband acceleration factor = 2.

A T2^*^-weighted field map was acquired with the following parameters: TE/T*R* = 10/1020ms; 40 slices; flip angle = 90°; in-plane matrix resolutio*n* = 80 × 80 mm; field-of-view = 200 × 200 mm; voxel size = 2.5 × 2.5 × 2.5 mm.

#### Preprocessing of rsfMRI Data

The rsfMRI data were preprocessed for seed-to-voxel whole-brain functional connectivity using the FMRIB Expert Analysis Tool (FEAT) within the FMRIB Software Library (FSL v5.0.11) (48). This included: removal of the first 5 volumes of the functional T2^*^-weighted scans; distortion-correction with field maps (which were prepared with the fsl_prepare_fieldmap tool); slice-timing correction; calculation of motion-related spatial realignment using Motion Correction FMRIB's Linear Image Registration Tool (MCFLIRT); removal of non-brain voxels using the Brain Extraction Tool (BET); and smoothing with a Gaussian kernel of 5 mm at full-width half-maximum (FWHM). Spatial realignment was based on 3 translational and 3 rotational dimensions, and these measures were later used to denoise the functional data.

#### Denoising and Registration of rsfMRI Data

Denoising was carried out in several steps. First, ICA-AROMA (ICA-based Automatic Removal of Motion Artifacts) (49) was applied to remove motion-related artifacts from the data using non-aggressive denoising. This method decomposes the data into independent components (IC) using independent component analysis, uses standardized features to identify components associated with head motion, and regresses these out of the data. ICA-AROMA is optimized for use after FSL FEAT preprocessing, and consistent with recommendations (49), ICA-AROMA was applied in native space after spatial smoothing but prior to high-pass filtering and further nuisance regression. ICA-AROMA identified a mean ± SD of 71.7 ± 9.3 components per subject, of which 55 ± 11% were classified as noise and removed. There was no between-group difference in the mean number of components identified or the mean percentage of components removed (Supplementary Table 1).

After this, the functional and anatomical scans were registered to MNI space using standard settings within FEAT. These included: linear registration between each participant's rsfMRI scan to their skull-stripped (using BET) anatomical T1-weighted scan, using FMRIB's Linear Image Registration Tool (FLIRT) with boundary-based registration, followed by nonlinear registration to the MNI152-2 mm space using FMRIB's Non-linear Image Registration Tool (FNIRT) with 12 degrees of freedom and a warp resolution of 10 mm. The transformations required for registration were calculated on the non-denoised rsfMRI data and applied to the ICA-denoised rsfMRI data.

Next, the MNI-registered scans and realignment parameters previously generated in FSL FEAT were imported into the Functional Connectivity Toolbox (CONN) v20b (http://www.conn-toolbox.org), implemented in the Statistical Parametric Mapping software package (SPM v.12; http://www.fil.ion.ucl.ac.uk/spm/software/spm12) and run on MATLAB (R2018a v.9.4; Mathworks, Nantick, MA) (50).

Given the sensitivity of rsfMRI data to motion, we sought to further measure and correct for frame displacement using the Artifact Rejection Toolbox (ART) within CONN. For this pediatric sample, outlier volumes were identified (for scrubbing) with a global signal *z-*value threshold = 9, and subjec*t-*motion threshold = 2 mm. Physiological noise associated with white matter (WM) and cerebrospinal fluid (CSF) was identified using aCompCor (51, 52), based on WM and CSF masks generated within CONN during tissue segmentation of the anatomical images. These masks were additionally eroded (erosion level 1) to minimize partial volume effects for each subject and were visually inspected.

Functional data were then denoised within CONN by regressing the following confounds: motion (based on realignment of functional scans during FSL preprocessing, comprising six motion dimensions with their first temporal derivatives, i.e., 12 parameters), physiological (comprising 5 WM and 5 CSF parameters), and scrubbing (for removal of outlier volumes identified with ART in 6/34 participants [range 2–17 volumes]). No participants were excluded based on motion thresholds. Linear detrending was performed and a high-pass filter of 0.008 Hz was applied to the data. The residual fMRI signals were used for whole-brain seed-to-voxel resting-state functional connectivity (rsFC) analysis.

We also assessed scan quality with respect to head motion during the rsfMRI scan. We extracted mean and maximum head motion per participant based on CONN'S ART composite motion framewise displacement (FD) measure. There was no difference between groups in either mean or maximum FD (Supplementary Table 1).

#### Seed-to-Voxel Resting-State Functional Connectivity

**Figure 1:**
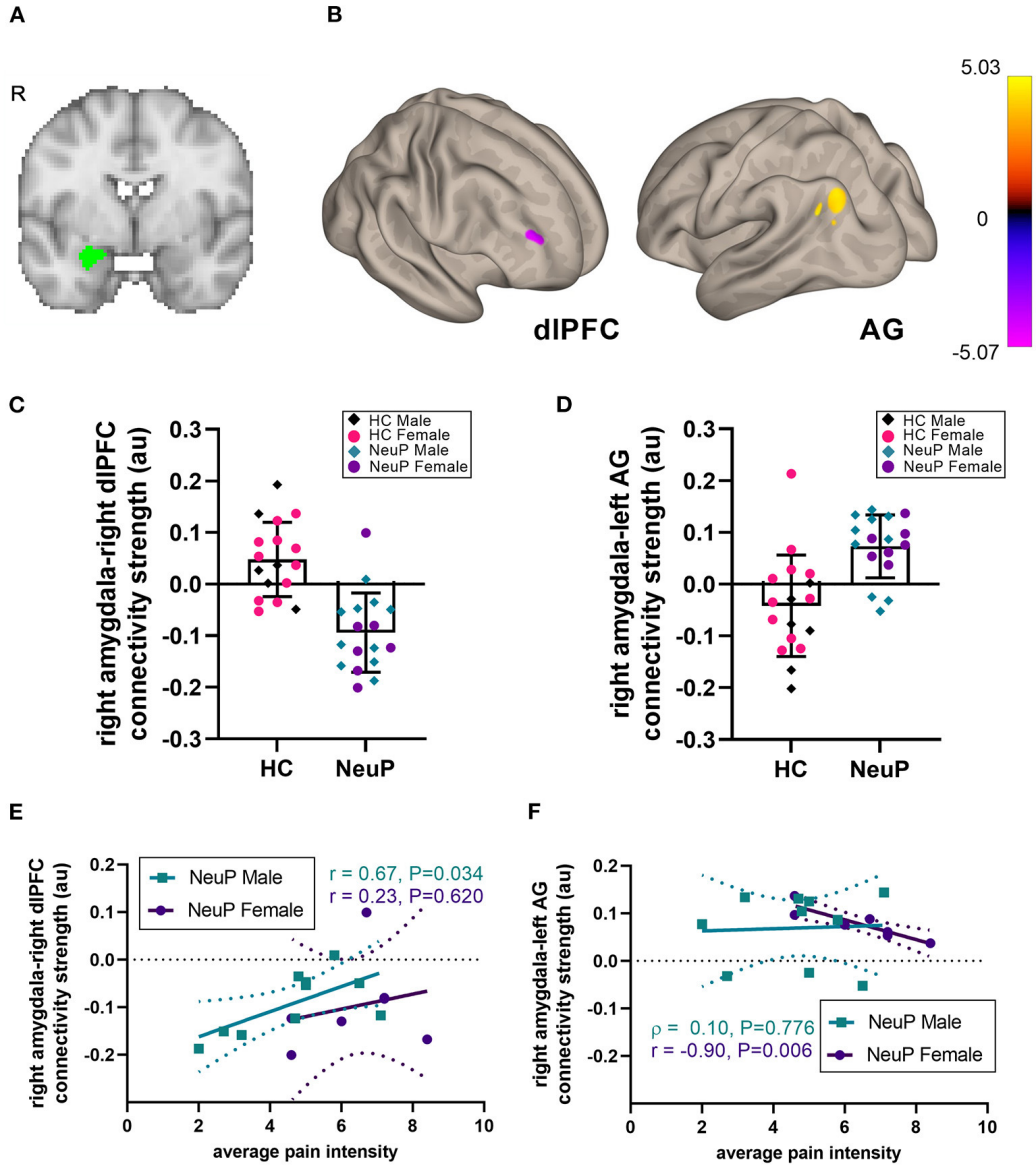
Right amygdalar rsFC network in adolescents with NeuP compared to control participants. **(A)** Location of the right amygdala seed. **(B)** We observed decreased amygdalar resting-state functional connectivity (rsFC) to the right dorsolateral prefrontal cortex (dlPFC), and increased rsFC to the left angular gyrus (AG), in patients compared to healthy controls (HC), significant at cluster-corrected P_FDR_ < 0.025. **(C)** Individual participant rsFC values between the right amygdala and the dlPFC, plotted separately for male and female NeuP patients and HC. **(D)** Individual participant rsFC values between the right amygdala and the left AG. **(E)** In male patients with NeuP, pain intensity positively correlated with the right amygdala-right dlPFC rsFC. **(F)** In female patients with NeuP, pain intensity negatively correlated with the right amygdala-left AG rsFC. **(C,D)** Data points represent individual values; bars represent mean [SD]. **(E,F)** Data points represent individual values; solid line represents regression line for correlation; dotted lines represent 95% confidence interval. au, arbitrary units; HC, healthy control; NeuP, neuropathic pain; R, right hemisphere; r, Pearson correlation; ρ, Spearman correlation.

We analyzed seed-to-voxel rsFC of the right and left amygdala to the rest of the brain in the CONN toolbox. We used the amygdala masks provided in CONN, which are based on the FSL Harvard-Oxford subcortical atlas (Figure 1A). To maximize association with other studies that use CONN, we used the default probability threshold (25%) for our amygdala masks.

We conducted two firs*t-*level, within-subject, fixed-effects seed-to-voxel analyses, one for each seed of interest: the right and left amygdalae. Each seed's timeseries was correlated with that of every other voxel in the brain. We then performed second-level random effects analysis to evaluate differences between healthy participants and those with NeuP for the right and left amygdala rsFC, while controlling for age. As our groups were not sex-matched, and because of reported sex differences in rsFC (53–58), sex-by-group regressors were used to compare NeuP vs. control participants. To enable group comparisons, voxelwise correlation coefficients were transformed to *z-*scores using Fisher's *r-*to-*z* transformation. Group differences between adolescents with NeuP and healthy controls were assessed with 2-sided parametric cluster-based statistics using the false-discovery rate (FDR). To correct for the two rsFC seeds we tested, we used a cluster size threshold of P_FDR_ < 0.025 (cluster-forming height threshold of *P* < 0.001). When group differences were identified, we also assessed whether rsFC in each group was significantly different from zero; we extracted the patients' *z-*transformed correlations for regions that were significantly different and conducted *post hoc* 1-sample *t-*tests.

In addition to analyzing seed-to-voxel rsFC for the right and left amygdala, we also explored whether there are hemispheric differences in amygdala rsFC between adolescents with NeuP and controls. To assess hemispheric differences within CONN, we added a between-sources contrast for the right and left amygdala. As before, group differences between NeuP and healthy controls were assessed with 2-sided parametric cluster-based statistics using the false-discovery rate. Maps were thresholded using cluster size at P_FDR_ < 0.025 (cluster-forming height threshold of *P* < 0.001).

#### *Post hoc* Correlation Analyses Between rsFC and Psychological Measures in Adolescents With NeuP

We next explored whether the differences in amygdala rsFC in patients with NeuP compared to healthy controls were related to the patients' average pain intensity and pain catastrophizing. We extracted the patients' *z-*transformed correlations for regions that were significantly different between groups and conducted *post hoc* correlation analyses with the patients' psychological measures. We also carried out within-sex *post hoc* correlation analyses with psychological measures. For significant within-sex correlations, we compared the correlation coefficients between males and females, using Fisher's *r*-to-*z*, and we assessed the effect of pain catastrophizing on the relationship between pain intensity and amygdala rsFC, using a Pearson partial correlation analysis.

### Statistical Analyses

Results relate to amygdala rsFC changes in a convenience sample of adolescents with NeuP and healthy controls. No *a priori* power calculation was performed, but previous studies have identified altered brain structure and function in similar samples of adolescents with CRPS (11–17).

Between-group rsFC comparisons were carried out within CONN. Analysis of clinical data, and *post hoc* correlations between clinical and rsFC measures, were performed with SPSS (v27; IBM, Portsmouth, UK). When assumptions of normality were not met, non-parametric tests were used. All tests were 2-tailed and assessed at *P* < 0.05. Analyses are based on available data, and sample size (*n*) is reported for comparisons based on fewer than 17 adolescents with NeuP.

Descriptive data are presented as mean ± SD or, if assumptions of normality were not met (assessed with Shapiro-Wilk test), as median (25th−75th percentile) [min–max]. Two-sample comparisons were conducted with Student's *t-*test or the Mann-Whitney-U test. Categorical data were compared with χ^2^ tests and exact 2-tailed *p-*values are reported. Bivariate correlations are reported as Pearson's r or Spearman's rho with bias-corrected 95% confidence intervals (CI). Effect sizes are reported for rsFC results based on η^2^ for Mann-Whitney tests; Phi for χ^2^ tests; and Hedge's g (related to Cohen's d but adapted for sample sizes <20) for independent samples *t-*tests (59, 60). Values of 0.01, 0.06, and 0.14 (η^2^), 0.1, 0.3, and 0.5 (phi), and 0.2, 0.5, and 0.8 (Hedge's g) are interpreted as small, medium, and large effects, respectively (59, 60).

Data were plotted with Prism v8 (GraphPad, San Diego, USA).

## Results

### Pain and Psychological Measures in Adolescents With NeuP

Average pain intensity in the last week was moderate-severe (Table 3), with females reporting higher levels of pain than males (t_15_ = 2.24, *P* = 0.041). On average, adolescents reported high pain catastrophizing scores (Table 3), with most either in the moderate (PCS-C 15-25; 40%) or high (PCS-C ≥26; 53%) range. Pain catastrophizing scores positively correlated with pain intensity (*r* = 0.58 [95%CI: 0.18, 0.84], *P* = 0.024, *n* = 15; Supplementary Figure 1).

**Table 3:** Pain intensity and interference ratings and pain catastrophizing scores in patients with NeuP.

	**NeuP (*n =* 17)**	**Male (*n =* 10)**	**Female (*n =* 7)**	**Group comparisons (male vs. female)**
**Pain intensity (0–10 cm VAS)**				
Average in last week	5.4 ± 1.7	4.7 ± 1.6	6.4 ± 1.4	*t*_15_ = 2.24, *P =* 0.041*
Worst in last week	7.4 ± 1.6	7.3 ± 1.6	7.6 ± 1.7	*t*_15_ = 0.36, *P =* 0.723
**Pain interference (0–10 cm VAS)**	7.7 (5.1-8.7) [1.6-9.3]	6.7 ± 2.4	8.0 (2.3-8.9) [2.0-8.9]	*Z* = 0.24, *P =* 0.807, *n =* 17
**Pain catastrophizing (0–52)**	27 ± 11; *n =* 15	25 ± 12; *n =* 8	29 ± 11; *n =* 7	*t*_13_ = 0.66, *P =* 0.519

*Normally distributed data are presented as mean ± SD and assessed with 2-sample t-test. Otherwise, data are presented as median (25th−75th percentile) [min-max] and assessed with Mann-Whitney test. Asterisks and bold text indicate significant correlations; brackets indicate number of subjects. Anchors for the pain intensity ratings are 0 (“no pain”) and 10 (“worst pain you can imagine”). Anchors for the pain interference ratings (“how much does pain interfere with your usual activities?”) are 0 (“not at all”) and 10 (“I can't do any of the things I want to do”). Pain interference ratings were completed at the time of recruitment to the study. NeuP, patients with neuropathic pain*.

### Right Amygdala rsFC Network

We compared seed-to-voxel rsFC of the amygdalae between our patient cohort and the control group (Figure 1; Table 4). Adolescents with NeuP had significantly reduced connectivity between the right amygdala and right dorsolateral prefrontal cortex (dlPFC) compared to the control group (cluster-corrected P_FDR_ < 0.025). Specifically, patients had negative amygdala-right dlPFC rsFC, whereas controls had positive connectivity between these regions (NeuP vs. HC mean ± SD: −0.09 ± 0.08 vs. 0.05 ± 0.07; Figure 1C). In both groups, amygdala-dlPFC rsFC was significantly different from zero [NeuP: *t*_(16)_ = 5.055, *P* < 0.0005. HC: *t*_(16)_ = 2.738, *P* = 0.015]. We also found significantly greater connectivity between the right amygdala and left angular gyrus (AG) in patients compared to controls (*P*_FDR_ < 0.025; Table 4). Specifically, patients had positive amygdala-left AG rsFC, whereas controls had negative connectivity between these regions (NeuP vs. HC median (25th−75th percentile) [min–max]: 0.09 (0.05–0.13) [−0.05–0.14] vs. −0.03 (−0.11–0.02) [−0.20–0.21]; Figure 1D). Amygdala-AG rsFC was significantly different from zero only in patients (NeuP: *t*_(16)_ = 4.968, *P* < 0.0005. HC: *t*_(16)_ = 1.755, *P* = 0.098).

**Table 4:** Right amygdala rsFC network in adolescents with NeuP.

**Brain Region**	**Peak MNI**			**Peak *T-*value**	**Number of voxels**
	**X**	**Y**	**Z**		
Left angular gyrus	−38	−68	32	5.03	88
Right dorsolateral prefrontal cortex	38	54	18	−5.07	73

*Peak MNI coordinates for brain regions with significantly altered right amygdala rsFC in adolescents with NeuP compared to healthy controls, significant at cluster-corrected threshold P_FDR_ < 0.025 and cluster-forming height threshold P < 0.001 (uncorrected). MNI, Montreal Neurological Institute*.

We found no group differences for the left amygdala, and no significant hemispheric (left vs. right amygdala) rsFC differences between adolescents with NeuP and healthy controls. Within-group amygdala rsFC is reported in Supplementary Tables 2–5.

### Relationship Between Right Amygdala rsFC and Pain Intensity in NeuP

To explore the relationship between rsFC differences in adolescents with NeuP and clinical characteristics, we performed *post hoc* correlations with average weekly pain intensity and pain catastrophizing. We did not observe whole-group correlations with pain intensity or pain catastrophizing (Table 5), perhaps reflecting sex-dependent or other within-cohort differences that are obscured by grouping. Therefore, and because reported pain intensity differed between males and females in this subgroup, we explored sex-dependent correlations. For the right amygdala-right dlPFC rsFC, we found a positive relationship with pain intensity only in males (*r* = 0.67, *P* = 0.034, *n* = 10; Figure 1E), indicating that stronger negative rsFC was associated with lower reported average pain intensity. For the right amygdala-left AG rsFC, we found a strong negative correlation with pain intensity only in females (*r* = −0.90, *P* = 0.006, *n* = 7; Figure 1F), indicating that stronger positive rsFC was associated with lower pain intensity. This correlation in females was also significantly stronger compared to the males (Fisher's *r*-to-*z*; *Z* = 2.42, *P* = 0.008). The correlation between right amygdala-right dlPFC connectivity and pain intensity was not significantly different in females compared to males (Fisher's *r*-to-*z*; *Z* = 0.92, *P* = 0.179). Pain catastrophizing did not correlate with rsFC (Table 5).

**Table 5:** Correlations between right amygdala rsFC and clinical measures in adolescents with NeuP.

**Brain region**			**Pain intensity (*n =* 17)**	**PCS-C (*n =* 15)**
Right dlPFC		*r*	*r =* 0.37, *P =* 0.149	*r =* 0.19, *P =* 0.498
		95% CI	−0.22, 0.77	−0.19, 0.54
	Males	r	***r =*** **0.67**, ***P =*** **0.034**, ***n =*** 10*	*r =* 0.02, *P =* 0.967, *n =* 8
		95% CI	−0.01, 0.98	−0.91, 0.94
	Females	r	*r =* 0.23, *P =* 0.620, *n =* 7	*r =* 0.30, *P =* 0.515, *n =* 7
		95% CI	−0.97, 1.00	−1.00, 1.00
Left AG		ρ	ρ = −0.27, *P =* 0.299	ρ = −0.01, *P =* 0.965
		95% CI	−0.77, 0.22	−0.67, 0.65
	Males	ρ	ρ = 0.10, *P =* 0.776, *n =* 10	ρ = 0.41, *P =* 0.317, *n =* 8
		95% CI	−0.89, 0.81	−0.62, 1.00
	Females	r	***r =*** **−0.90**, ***P =*** **0.006**, ***n =*** 7*	*r =* −0.61, *P =* 0.146, *n =* 7
		95% CI	−1.00, −0.68	−0.99, −0.09

*Values are Pearson's r for normally distributed data and Spearman's rho (ρ) otherwise, with bias-corrected 95% confidence intervals (CI); asterisks and bold text indicate significant correlations; brackets indicate number of subjects. AG, angular gyrus; dlPFC, dorsolateral prefrontal cortex; PCS-C, Pain Catastrophizing Scale, child version*.

Next, given that the dlPFC and AG participate in the frontoparietal network, suggesting involvement in cognitive-affective processing, and that pain intensity correlated with pain catastrophizing (Supplementary Figure 1), we explored whether negative pain cognitions, captured by the PCS-C, modulated the relationship between rsFC and reported pain intensity. Partial correlation analyses showed that the correlation between pain intensity and right amygdala-right dlPFC rsFC in the males was still significant after controlling for PCS-C (r_PCS−C_ = 0.83, *P* = 0.021, *n* = 8, df = 5), and likewise for the correlation between pain intensity and right amygdala-left AG rsFC in the females (r_PCS−C_ = −0.84, *P* = 0.039, *n* = 7, df = 4).

## Discussion

In adolescents with moderate-severe peripheral NeuP, we identified significantly different amygdala rsFC with the dlPFC and AG, which participate in a cognitive control network (the frontoparietal network; FPN) (61, 62), suggesting altered cognitive-affective processing. Specifically, there was stronger negative right amygdala-right dlPFC rsFC, and stronger positive rsFC between the right amygdala and left AG, compared to age-matched adolescents. Exploratory within-sex correlations showed that rsFC differences in adolescents with NeuP were associated with lower pain intensity, suggesting that the observed connectivity differences may contribute to inhibitory modulation.

### Amygdala rsFC Differences Associated With Pain Modulatory Circuits

The amygdala is well-placed to modulate pain experience (63, 64). It receives direct nociceptive input via the spino-parabrachio-amygdaloid tract (65–67), afferent thalamic inputs (68), and has direct pain modulatory projections to the periaqueductal gray (20, 69, 70). Evidence from rodent studies suggests both anti- and pro-nociceptive functions for the amygdala (71, 72). In human neuroimaging studies, experimental noxious stimuli frequently elicit increased amygdalar responses in both healthy adults and those with clinical pain conditions (23). In healthy adults, amygdala rsFC was associated with individual differences in emotional pain modulation (73). Studies in both adolescents and adults with chronic pain have also identified abnormal amygdalar connectivity with nociceptive processing and pain modulatory regions (13, 28–30).

Our first key finding was that the amygdala had stronger negative rsFC to the dlPFC, compared to controls. The dlPFC is a key node of the FPN and other networks (74), and has a role in cognitive and emotional control. It is frequently activated in experimental pain studies, and is often shown to have abnormal structure and function in chronic pain populations (74, 75). In task fMRI studies, the association of the dlPFC with empathetic modulation of pain (76), pain inhibition, perceived control of pain, and pain anticipation (74), suggest a role in to*p-*down modulation of pain. Furthermore, its involvement in multiple resting state networks suggests several mechanisms through which it could achieve such modulatory functions, including reducing emotional reactivity to pain via limbic circuitry (74), such as its connectivity with the amygdala. Accordingly, a task fMRI study found that amygdala-dlPFC connectivity was related to individual differences in emotional regulation of pain in healthy adults (77). Similarly, a resting state fMRI study found that this connectivity was involved in pain modulation through guided music listening in adults with fibromyalgia (78). Finally, this connectivity at rest was also correlated to increased pain-related fear in adolescents with CRPS (13). Consistent with these studies, the association between amygdala rsFC differences and reduced pain intensity in our cohort indicates a role in pain-related modulation.

Our second key finding was that those with NeuP had stronger positive rsFC between the right amygdala and left AG. Similarly to the dlPFC, the AG, a subregion of the posterior parietal cortex, is a key node of the FPN and other intrinsic brain networks (79). Previous fMRI studies involving experimental manipulations related to regulation of pain-related emotion identify AG responses during viewing of negative (vs. neutral) images (80) and during listening to unempathetic (vs. neutral) comments (76) in healthy adults experiencing experimental (thermal) pain. Another study identified AG responses during reappraisal of painful (vs. non-painful) scenarios that downregulate empathy for pain in healthy adults (81). In adults with fibromyalgia, music-induced analgesia was associated with reduced AG rsFC to several nodes of the default mode network (DMN) (78). Notably, the AG is also a key node of the DMN, which is involved in introspection and memory (82, 83). Therefore, changes in connectivity between the AG and DMN may reflect distraction or memory/attention-related processes (9).

Altered FPN rsFC has also been associated with expectancy-induced modulation of pain in healthy adults (84). In adults with chronic lower back pain, increased rsFC between bilateral amygdala and the FPN positively correlated with pain catastrophizing and pain intensity, suggesting altered cognitive-emotional interactions for pain modulation (28).

### Association of Amygdala rsFC With Reduced Pain Intensity

Previous studies report increased amygdala FC in patients with chronic pain that correlates with poorer function, including greater pain intensity (28, 30), greater pain catastrophizing (28), and increased pain-related fear (13). In contrast, we found amygdala rsFC to FPN nodes correlated with reduced pain intensity, which may reflect a resilience mechanism rather than vulnerability. This may relate to differences in our cohort compared to others (13, 28, 30), including sex and age range, pain disorders investigated, and pain/cognitive characteristics (pain duration, pain catastrophizing). Interestingly, another study reported altered amygdalar rsFC to regions including the dlPFC and AG that was similarly associated with improved function, specifically headache reduction following cognitive behavioral therapy in adolescents with migraine (9). Similarly, amygdala-dlPFC rsFC was associated with music-induced analgesia in adults with fibromyalgia (78).

Although our sample is small, the finding that stronger *negative* amygdala-dlPFC rsFC is associated with reduced pain intensity in males with NeuP suggests that the dlPFC may be inhibiting limbic circuitry. Similarly, in a task-fMRI study of controllable vs. uncontrollable pain in healthy adults, increased negative connectivity between the bilateral dlPFC and pain-related regions during the controllable (thermal) pain task was associated with reduced pain, suggesting that the dlPFC suppressed activity in these regions (85). In pain-free adults, placebo analgesia was associated with increased dlPFC and orbitofrontal activation during pain anticipation, and this increased activity correlated with reduced activity in sensory regions during the pain phase, suggesting an inhibitory effect on sensory regions to reduce pain intensity (86).

Interestingly, although patients had stronger negative amygdala-dlPFC rsFC compared to controls, in male patients stronger negative rsFC was associated with reduced pain; likewise, patients had stronger positive amygdala-AG rsFC, but in female patients stronger positive rsFC was associated with reduced pain. Similar findings have been reported in other pediatric studies: One study assessing structural connectivity in adolescents with chronic headaches found that patients had increased fractional anisotropy (FA) in the cingulum (a corticolimbic tract) compared to controls, but within the patient group, those with lower FA had a greater headache frequency, suggesting greater disease progression (87). A second study reported that youth with chronic pain (including musculoskeletal, neuropathic, and visceral pain) had increased rsFC between the amygdala and inferior parietal lobe (IPL) compared to controls, but in the patients stronger positive rsFC was associated with lower pain catastrophizing and reduced differential fear associated with a threa*t-*safety conditioning task (10). In the controls, catastrophizing was not associated with amygdala-IPL rsFC, suggesting that the amygdala rsFC and its association with pain catastrophizing may differ in patients with chronic pain, and may be protective (10). In our study, we were not able to assess the relationship between amygdala rsFC and pain intensity in our control participants, because they did not have chronic clinical pain and did not experience an experimental pain task during scanning. The positive amygdala-dlPFC rsFC observed in the controls could relate to other factors unrelated to current pain, as these limbic-cognitive networks are involved in many functions.

Overall, associations with reduced pain intensity in our cohort of adolescents with NeuP could indicate strengthening over time of circuitry associated with pain inhibition, to compensate for chronically increased nociceptive input, or with individual ability to disengage from the pain when mind-wandering (88) during the rsfMRI scan or watching a movie during the 15–20 mins preceding the rsfMRI scan (see Methods). Alternatively, individual differences in amygdala connectivity may contribute to differences in pain intensity. Longitudinal investigations that include a pain manipulation in pain-free participants and/or patients with chronic pain, or neuroimaging before and following therapy or resolution of pain symptoms in patients, are needed to further disentangle the underlying mechanisms of the observed relationships.

In our study, pain catastrophizing did not correlate with amygdala rsFC (Table 5) and did not moderate the correlations with pain intensity, suggesting that reduced maladaptive cognitions did not contribute to the association with reduced pain intensity. However, pain experience is shaped by multiple factors, and our small sex-dependent samples preclude strong conclusions. Lack of association with pain catastrophizing could also reflect the high proportion (93%) reporting moderate-high PCS-C scores, consistent with a study that reported altered frontolimbic rsFC in adolescents with chronic pain and high PCS-C scores, compared to those with low PCS-C scores and to controls (89).

### Amygdala Lateralization

Here we observed group differences in rsFC for the right amygdala but not the left. Previous studies in human participants reported amygdala rsFC differences and associations with poorer function mainly in the left amygdala (with a few differences in the right amygdala) (13) or in both hemispheres (28, 30). It is difficult to draw conclusions about lateralization of human amygdala function, as observed hemispheric differences may partly reflect confounding factors, such as stimulation side, stimulation modality, and demographics (e.g., sex) across studies (23, 71). Further research is required to explore possible lateralization in pediatric chronic pain cohorts.

### Clinical Relevance

Mechanisms, management, and prognosis of NeuP differ from other types of persistent pain (1), and NeuP may involve disease-specific brain alterations (90). Furthermore, presentation, prevalence, and causes of NeuP in adolescents differ from those in adults (1), and laboratory studies indicate that pain mechanisms change with age (3). Therefore, identifying relationships between reported pain experience and pain circuitry across different types of chronic pain may improve understanding and inform management. Our cohort of adolescents with NeuP reported significant pain and high levels of pain catastrophizing and emotional distress (39), and psychological factors are important components of a biopsychosocial formulation and multidisciplinary management plan (91). While alterations in cognitive-affective circuitry and increased amygdala rsFC have also been identified in other chronic pain cohorts (13, 28) we found associations with reduced pain. As there is increasing awareness of the impact of resilience on neural circuitry and pain-related outcomes (92), there is an ongoing need to identify mechanisms and factors associated with inhibitory modulation.

### Limitations

The sample size is relatively small, but comparable to pediatric CRPS MRI studies (11–14). Variations in pain site (location and side) can impact brain processes; future research in larger samples should account for such variability. The association of amygdala rsFC with reduced pain intensity is correlational and requires further investigation with longitudinal studies in larger samples. Control participants were not perfectly matched for sex, and specific information related to pain, mood, and other characteristics (ethnicity, socioeconomic status) was not collected. We did not consider gender, pubertal stage, or phase of the menstrual cycle. While suggestive of sex-dependent differences in circuits modulating reported pain intensity in males and females, a larger sample is required to confirm this. As this cross-sectional cohort was recruited from a chronic pain clinic, duration of pain and pharmacotherapy at the time of the MRI scan were variable. Physiological monitoring was not performed during scans, so physiological noise was not directly regressed out; however, we used computational approaches including aCompCor (which relies on noise components in non-gray matter regions) to correct for physiological noise. Watching a movie prior to acquisition of rsfMRI scans, and keeping eyes closed vs. open or fixated on an object, can impact resting state networks in different ways. Given our pediatric and clinical sample, where head motion and movement artifact are more common and may necessitate removal of significant amounts of data and affect interpretation of results, we used strategies for making participants more comfortable during the scan [see discussion in Verriotis et al. (41)].

## Conclusions

Adolescent chronic NeuP is associated with high levels of pain and pain catastrophizing (39). Although a small sample, our results reflect an understudied pediatric cohort with chronic peripheral NeuP and highlight rsFC differences relative to controls within pain modulatory circuits. Understanding the brain mechanisms and clinical, pathophysiological, and psychosocial factors associated with pain modulation, potential sexual dimorphism in central pain mechanisms, and additional sources of individual variability in pediatric chronic NeuP, may ultimately contribute to improved patient stratification and management.

## Data Availability Statement

The datasets presented in this article are not readily available because sharing of any MRI data would be subject to our institutional approval of anonymization and data governance requirements. Requests to access the datasets should be directed to MV, madeleine.verriotis@ucl.ac.uk.

## Ethics Statement

The studies involving human participants were reviewed and approved by National Health Service Research Ethics Committee. Written informed consent to participate in this study was provided by the participants' legal guardian/next of kin.

## Author Contributions

MV and SMW contributed to conception of the study. MV, JP, KKS, CAC, MM, and SMW contributed to design of the study. MV, CS, JP, and SMW collected patient data. MV, CS, LJA, MM, and SMW contributed to analysis and/or interpretation. MV drafted the manuscript. All authors contributed to manuscript revision, read, and approved the submitted version.

## Funding

This research was supported by funds from Great Ormond Street Hospital Children's Charity Research Awards W1071H, W1071I (SMW) and a University College London—University of Toronto Joint Research Project and Exchange Activities Award (CAC, MM, MV, and SMW). CS is supported by a Child Health Research Charitable Incorporated Organisation (CHR CIO) Great Ormond Street Institute of Child Health—University of Toronto PhD Studentship. LJA is supported by Canadian Institutes of Health Research (CIHR) Frederick Banting and Charles Best Doctoral Research Award. MM is supported by a University of Toronto Centre for the Study of Pain and the Bertha Rosenstadt Endowment Fund. Research at Great Ormond Street Hospital NHS Foundation Trust and UCL Great Ormond Street Institute of Child Health is supported by the NIHR Great Ormond Street Hospital Biomedical Research Centre.

## Author Disclaimer

The views expressed are those of the authors and not necessarily those of the NHS, the NIHR or the Department of Health.

## Conflict of Interest

The authors declare that the research was conducted in the absence of any commercial or financial relationships that could be construed as a potential conflict of interest.

## Publisher's Note

All claims expressed in this article are solely those of the authors and do not necessarily represent those of their affiliated organizations, or those of the publisher, the editors and the reviewers. Any product that may be evaluated in this article, or claim that may be made by its manufacturer, is not guaranteed or endorsed by the publisher.

## Abbreviations

AG: angular gyrus
CRPS: complex regional pain syndrome
dlPFC: dorsolateral prefrontal cortex
DMN: default mode network
FDR: false discovery rate
FPN: frontoparietal network
GOSH: Great Ormond Street Hospital
HC: healthy control
IPL: inferior parietal lobe
NeuP: neuropathic pain
NHS: National Health Service
PCS-C: Child version of the Pain Catastrophizing Scale
PROMs: patien*t-* and paren*t-*reported outcome measures
QST: quantitative sensory testing
rsFC: resting-state functional connectivity
rsfMRI: resting-state functional magnetic resonance imaging
VAS: visual analog scale.
