# Supplementary material for: Amygdalar Functional Connectivity Differences Associated With Reduced Pain Intensity in Pediatric Peripheral Neuropathic Pain
Source: Front Pain Res (Lausanne). 2022 May 27;3:918766. doi: 10.3389/fpain.2022.918766 (PMC9184677; doi:10.3389/fpain.2022.918766)
Supplement: Supplementary file 1 [file Data_Sheet_1.pdf]

Supplementary Material

1 Supplementary Figures

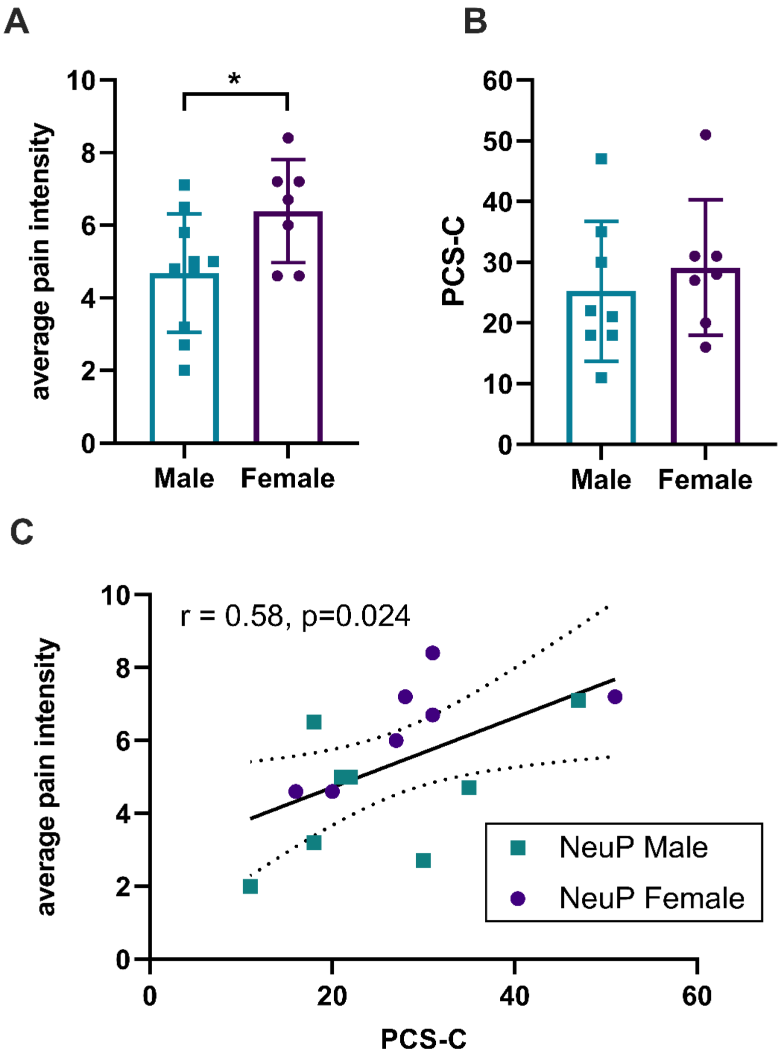

Supplementary Figure 1. Pain ratings and pain catastrophizing in adolescents with NeuP. (**A**) Individual participant ratings for average pain intensity in the last week, plotted separately for male and female NeuP patients. (**B**) Individual participant pain catastrophizing scores, plotted separately for male and female NeuP patients. (**C**) Pain catastrophizing correlated positively with average pain intensity in the last week; n=15. ***(A-B)*** Data points represent individual values; bars represent mean [SD]; asterisk indicates significance at P<0.05. ***(C)*** Data points represent individual values; solid lines represent regression line for correlation; dotted lines represent 95% confidence interval. *Abbreviations:* NeuP, neuropathic pain; PCS-C, Pain Catastrophizing Scale, child version**.**

2 Supplementary Tables

Supplementary Table 1. Comparative ICA-AROMA independent component and head motion data for patients and control participants.

	NeuP (n=17)	HC (n=17)	Group Comparisons	Effect size
Independent Components				
Mean ICs	72.5±10.2	70.0±8.7	$t_{(32)}$=0.473, P=0.640	0.158
Mean %noise ICs	57±9	53±13	$t_{(32)}$=0.918, P=0.365	0.307
Head motion				
Mean FD (mm)	0.12 (0.11-0.16) [0.08-0.28]	0.12 (0.10-0.15) [0.06-0.24]	Z=0.60, P=0.547	0.011
Maximum FD (mm)	0.66 (0.36-1.04) [0.22-1.76]	0.44 (0.30-2.38) [0.13-9.10]	Z=0.36, P=0.718	0.004

Data = mean±SD or, if assumptions of normality were not met, data = median (25th-75th percentile)[min-max]. Effect size = η^2 for Mann-Whitney tests and Hedge's g for independent samples t-tests. Standardised test statistic (Z-value) is reported for Mann-Whitney test. *Abbreviations*: FD, framewise displacement (in millimetres); HC, healthy control participants; IC, independent component; NeuP, patients with neuropathic pain.

Supplementary Table 2. Right amygdala rsFC network within adolescents with NeuP.

Brain Region	Peak MNI			Peak *T*-value	Number of voxels
	X	Y	Z		
Positive rsFC					
Right Amygdala	26	2	-18	43.40	2402
Right Parahippocampal Gyrus	18	0	-20	30.47	
Right Hippocampus	22	-8	-14	24.80	
Left Amygdala	-24	-6	-10	16.43	1409
Left Hippocampus	-28	-20	-18	12.47	
Left Amygdala	-18	0	-16	12.24	
Left Inferior Temporal Gyrus	-46	-50	-24	5.77	247
Left Fusiform Cortex	-36	-42	-26	5.74	
Left Fusiform Cortex	-40	-42	-18	5.45	
Left Primary Motor Cortex	-46	-16	56	6.20	127
Left Primary Somatosensory Cortex	-48	-22	62	5.45	
Right Middle Temporal Gyrus	52	-48	-2	6.97	105
Right Inferior Temporal Gyrus	54	-42	-14	6.21	
Posterior Midcingulate Cortex	4	-2	42	7.57	75
Anterior Midcingulate Cortex	6	6	34	6.28	
Right Rectus Gyrus	8	48	-18	5.32	67
Left Frontal Medial Orbital Cortex	-6	40	-12	5.07	
Left Frontal Medial Orbital Cortex	0	54	-6	4.78	
Left Primary Somatosensory Cortex	-66	-14	20	6.33	65
Subcallosal Cortex	4	6	-2	5.91	64
Subcallosal Cortex	2	8	-16	5.85	
Right Middle Temporal Gyrus/Inferior Temporal Gyrus	64	-34	-18	8.92	59
Right Primary Motor Cortex	46	-10	38	5.94	49
Left Angular Gyrus	-36	-66	30	6.82	45
Left Angular Gyrus	-34	-74	36	5.61	
Right Premotor Cortex	58	2	28	5.12	39
Right Primary Motor Cortex	60	-2	18	4.93	
Left Paracentral Lobule	-6	-36	56	5.69	36
Negative rsFC					
Right Dorsolateral Prefrontal Cortex	36	52	24	-5.16	45
Right Dorsolateral Prefrontal Cortex	32	46	18	-5.01	
Right Angular Gyrus	54	-48	34	-5.80	39

Peak MNI coordinates for brain regions with significant right amygdala rsFC in adolescents with NeuP, significant at cluster-corrected threshold $P_{FDR}<0.025$ and cluster-forming height threshold $P<0.001$ (uncorrected). *Abbreviations*: MNI, Montreal Neurological Institute.

Supplementary Table 3. Right amygdala rsFC network within HC adolescents.

**Brain Region**	**Peak MNI**			**Peak *T*-value**	**Number of voxels**
	**X**	**Y**	**Z**		
Positive rsFC					
Right Hippocampus	28	-6	-16	23.13	1904
Right Amygdala	28	2	-20	13.90	
Right Hippocampus/Parahippocampal Gyrus	18	-12	-18	12.89	
Left Hippocampus	-22	-10	-16	9.98	771
Left Hippocampus	-14	-8	-14	8.73	
Left Parahippocampal Gyrus/Amygdala	-20	0	-28	8.72	
Right Superior Temporal Gyrus	64	-4	2	6.50	210
Right Superior Temporal Gyrus/Middle Temporal Gyrus	62	-2	-10	6.42	
Right Ventral Premotor Cortex	60	0	14	5.82	
Left Primary Somatosensory Cortex	-60	-16	38	6.51	158
Left Primary Somatosensory Cortex	-58	-24	40	4.40	
Right Superior Temporal Pole	48	24	-24	7.10	124
Right Middle Temporal Pole	52	18	-30	4.93	
Right Primary Somatosensory Cortex	62	-10	36	5.48	113
Right Primary Motor Cortex	54	-6	34	5.25	
Right Primary Somatosensory Cortex/Primary Motor Cortex	64	-2	32	4.52	
Left Premotor Cortex	-46	-10	32	5.60	48
Right Temporooccipital Inferior Temporal Gyrus	46	-56	-14	6.30	45

Peak MNI coordinates for brain regions with significant right amygdala rsFC in HC adolescents, significant at cluster-corrected threshold $P_{FDR}<0.025$ and cluster-forming height threshold $P<0.001$ (uncorrected). *Abbreviations*: MNI, Montreal Neurological Institute.

Supplementary Table 4. Left amygdala rsFC network within adolescents with NeuP.

**Brain Region**	**Peak MNI**			**Peak *T*-value**	**Number of voxels**
	**X**	**Y**	**Z**		
Positive rsFC					
Left Amygdala	-18	0	-18	38.33	2940
Left Hippocampus	-26	-4	-20	31.10	
Left Parahippocampal Gyrus/Fusiform Cortex	-26	-2	-30	16.30	
Right Hippocampus	30	-20	-14	13.05	1295
Right Parahippocampal Gyrus	16	-2	-18	12.74	
Right Amygdala	28	-6	-14	10.87	
Left Primary Somatosensory Cortex	-64	-8	16	7.41	212
Left Primary Somatosensory Cortex/Primary Motor Cortex	-60	-4	30	7.23	
Left Somatosensory Cortex	-68	-10	24	5.23	
Right Cerebellum/Fusiform Cortex	36	-42	-26	6.23	191
Right Cerebellum	14	-52	-18	5.98	
Right Cerebellum	34	-34	-30	5.32	
Anterior Midcingulate Cortex	-2	-2	36	10.52	125
Anterior Midcingulate Cortex	6	4	36	9.07	
Left Superior Temporal Gyrus	-54	-6	-4	7.06	86
Left Middle Temporal Gyrus	-62	-14	-2	5.59	
Left Middle Temporal Gyrus	-58	-12	-10	5.01	
Left Primary Motor Cortex	-42	-18	68	5.87	85
Left Primary Motor Cortex	-42	-14	60	5.42	
Left Primary Motor Cortex	-40	-10	52	5.08	
Right Lateral Orbitofrontal Cortex	30	30	-16	6.59	43

Peak MNI coordinates for brain regions with significant left amygdala rsFC in adolescents with NeuP, significant at cluster-corrected threshold $P_{FDR}<0.025$ and cluster-forming height threshold $P<0.001$ (uncorrected). *Abbreviations*: MNI, Montreal Neurological Institute.

Supplementary Table 5. Left amygdala rsFC network within HC adolescents.

**Brain Region**	**Peak MNI**			**Peak *T*-value**	**Number of voxels**
	**X**	**Y**	**Z**		
Positive rsFC					
Left Hippocampus/Amygdala	-26	-6	-18	26.73	2095
Left Hippocampus/Amygdala	-18	-6	-12	18.21	
Left Hippocampus	-26	-14	-10	17.59	
Right Hippocampus	24	-14	-10	10.32	887
Right Hippocampus/Parahippocampal Gyrus	30	-12	-20	9.48	
Right Amygdala	26	0	-10	8.06	
Left Fusiform Cortex	-40	-46	-22	6.00	87
Left Fusiform Cortex	-30	-46	-20	4.54	
Left Fusiform Cortex/Inferior Temporal Gyrus	-46	-52	-18	4.52	
Left Middle Temporal Gyrus	-54	-60	0	5.85	83
Left Middle Temporal Gyrus/Lateral Occipital Cortex	-46	-64	2	5.50	
Left Inferior Temporal Gyrus	-42	-60	-6	7.23	64
Left Fusiform Cortex	-40	-50	-6	5.73	
Pregenual Cingulate Cortex	-6	36	2	7.04	48
Pregenual Cingulate Cortex	2	34	12	4.58	
Left Lateral Orbitofrontal Cortex	-40	28	-16	4.84	46
Left Lateral Orbitofrontal Cortex	-42	36	-20	4.84	

Peak MNI coordinates for brain regions with significant left amygdala rsFC in HC adolescents, significant at cluster-corrected threshold $P_{FDR}<0.025$ and cluster-forming height threshold $P<0.001$ (uncorrected). *Abbreviations*: MNI, Montreal Neurological Institute.
